# Biomarkers of Apoptosis and Systemic Inflammation in Patients with COVID-19 Undergoing Targeted Anticoagulant Therapy

**DOI:** 10.3390/life16101693

**Published:** 2026-10-09

**Authors:** Roman Kalinin, Igor Suchkov, Andrey Agapov, Vladislav Povarov, Nina Mzhavanadze

**Affiliations:** Department of Cardiovascular, Endovascular Surgery, and Diagnostic Radiology, Ryazan State Medical University, 390026 Ryazan, Russia; kalinin-re@yandex.ru (R.K.);

**Keywords:** biomarkers, apoptosis, systemic inflammation, COVID-19 targeted anticoagulant therapy

## Abstract

Background: We assessed apoptosis biomarkers alongside systemic inflammatory indices in patients diagnosed with COVID-19 undergoing targeted anticoagulant therapy. Methods: A prospective cohort study involved a total of 370 patients hospitalized with COVID-19 from 2021 to 2022. One hundred ninety patients with COVID-19 received low-molecular-weight heparins, LMWH (group 1), one hundred twenty-three patients received unfractionated heparin, UFH (group 2), and fifty-seven patients received direct oral anticoagulants, DOAC (group 3). The diagnostic protocol included assessment of clinical and anamnesis data, evaluation of biomarkers of apoptosis (phosphatidylserine and calreticulin) and systemic inflammation (C-reactive protein, ferritin, procalcitonin) alongside duplex venous ultrasonography of the lower extremities executed to screen for venous thromboembolic complications. Results: The LMWH regimen elicited a substantial chronological surge in phosphatidylserine concentrations, demonstrating an 18.4% increment from baseline parameters (*p* = 0.012) in COVID-19 patients. Baseline phosphatidylserine levels above 62.75 pg/mL were a protective factor associated with attenuating the odds of developing an episode of venous thromboembolism by 1.033 (*p* = 0.02). Conclusions: Phosphatidylserine can be used as a prognostic measure to evaluate the odds of developing a VTE episode. LMWHs exert more potent pleiotropic anti-inflammatory and systemic anticoagulant activities within this infectious framework compared to alternative ACT protocols.

## 1. Introduction

Anticoagulant therapy (ACT) is an established route to prevent or treat venous thromboembolism [1]. However, efficient targeted ACT may be attenuated due to a number of causes triggering ongoing procoagulant hemostasic shift. These destabilizing mechanisms are frequently operative during acute systemic infections, septic states, and, notably, within the pathophysiological framework of COVID-19. Consequently, hypercoagulability and refractory thrombosis under these conditions reinforce the critical necessity to rigorously investigate and refine ACT protocols in the context of infectious virus-induced coagulopathies [2].

Viral pathologies are inherently linked to severe hemostatic disruptions of the coagulation network. Operating in tandem with systemic inflammatory responses, these infectious agents can trigger a spectrum of clinical catastrophes, ranging from localized thrombosis and acute exsanguinating hemorrhage to the complex, simultaneous manifestation of both pathological processes [3]. By directly inducing endothelial injury, viruses stimulate the hypersecretion of an extensive pool of pro-inflammatory cytokines. This cytopathic cascade sequentially drives the upregulation of blood coagulation factors, which in turn facilitates platelet and monocyte aggregation within the vascular intimacy alongside the high-density expression of tissue factor, ultimately culminating in microvascular or macrovascular thrombus architecture assembly [4]. Given the critical, life-threatening risk of thrombosis during COVID-19 progression, the clinical necessity of targeted antithrombotic management has attained global consensus; nevertheless, significant therapeutic limitations and translational gaps remain to be resolved within current consensus protocols [5].

Elucidating the underlying molecular pathways that drive coagulopathy progression during acute infectious conditions remains paramount for the design of targeted therapeutic strategies; thus, identifying novel biomarkers that perturb the coagulation equilibrium constitutes a highly relevant research mandate [6,7]. A promising vector within this domain encompasses the investigation of apoptosis mechanisms. During inflammatory responses orchestrated within the framework of immunothrombosis, reciprocal interactions between neutrophils and platelets prompt robust platelet activation, monocytic tissue factor expression, and the subsequent shedding of microparticles and phosphatidylserine [8]. Systemic inflammation actively triggers the activation and apoptotic decay of blood cells, a cytopathic process that facilitates microvesicle release and the translocation of membrane phospholipids—specifically phosphatidylserine—to the outer leaflet of the cellular plasma membrane, thereby serving as a catalytic template for thrombin generation and localized clot architectural assembly [9]. Furthermore, this phospholipid externalization onto the surface of apoptotic cells functions as an evolutionary “eat-me” signaling pattern recognized by specific phagocytic receptors. The subsequent engulfment of apoptotic remnants by phagocytes effectively clears cellular debris, thereby preventing secondary pro-inflammatory injury within the host organism [10]. Under apoptotic stress constraints, cells exhibit an elevated expression of calreticulin, a chaperone that binds with phosphatidylserine in a calcium–mediated was [11]. Calreticulin is primarily localized in the endoplasmic reticulum and plays a key role in calcium homeostasis and subsequently hemostasis [12,13]. Some studies show that calreticulin may bind to coagulation factors and inhibit intravascular thrombosis in experimental animal models [14].

A defining hallmark of coronavirus-induced coagulopathy is immunothrombosis, a process in which systemic hyperinflammation acts as a primary driving determinant. Upon cellular invasion by SARS-CoV-2, pro-inflammatory cytokines trigger a robust upregulation of cell adhesion molecules on the luminal surface of endothelial cells, specifically P-selectin and E-selectin, which facilitate the rapid recruitment and tethering of circulating leukocytes to the foci of vascular injury and inflammation [15]. Concurrently, bound platelets undergo robust activation under the cytopathic influence of SARS-CoV-2 and shed extracellular vesicles enriched with the high-mobility group box 1 (HMGB1) protein. This microvesicular cargo induces the deployment of neutrophil extracellular traps (NETs)—complex web-like structures that mechanically trap circulating blood cells and plasma coagulation factors, thereby reinforcing localized thrombus structural stability [16].

Activated platelets bridge with immune cells, thus unifying prothrombotic and inflammatory routes leading to a thrombotic event [17]. Patients with severe and critical COVID-19 requiring ICU treatment were shown to exhibit a high tendency toward platelet-to-leukocyte aggregate formation [18].

Given the demonstrated capacity of phosphatidylserine and calreticulin to bridge the intricate networks of thrombotic and inflammatory cascades, alongside their direct involvement in blood cell apoptotic pathways, we systematically investigated the chronological dynamics of these specific indicators and acute-phase inflammatory reactants. This evaluation was conducted within a defined cohort of patients undergoing distinct targeted anticoagulant strategies designed for the prophylaxis of venous thromboembolism (VTE). We aimed to evaluate the chronological dynamics of apoptosis biomarkers, specifically phosphatidylserine and calreticulin, alongside systemic inflammatory indices in patients diagnosed with COVID-19 undergoing targeted anticoagulant therapy.

## 2. Materials and Methods

*Study Design and Participant Stratification*

A prospective cohort study was executed, enrolling a total of 370 patients undergoing targeted anticoagulant therapy (ACT) for the management of laboratory-confirmed and instrumentally verified SARS-CoV-2 infection, from 2021 to 2022. Table S1 in the Supplementary Materials Section provides a STROBE Statement with a checklist of items that should be included in reports of cohort studies.

*Eligibility Criteria*

Inclusion Criteria: Male or female patients aged ≥ 18 years, with documented COVID-19 diagnosis confirmed via standardized laboratory molecular testing and neuroimaging or thoracic computed tomography, and signed informed consent to participate in the study protocol prior to enrollment.

Exclusion Criteria: Age under 18 years; pregnancy or active lactation period; moribund or agonizing physical status necessitating immediate admission to the intensive care unit (ICU) upon arrival; pre-existing or baseline thrombocytopenia; and any definitive absolute contraindications to systemic anticoagulant administration. Thrombocytopenia was defined as platelet count less than 150 × 10^9^/L or 150,000 cells/µL.

*Ethical Considerations and Trial Registration*

The active study protocol was rigorously reviewed and approved by the Local Ethics Committee (Protocol No. 3, dated 11 October 2021). The clinical trial was prospectively registered on the ClinicalTrials.gov registry platform with the unique repository identifier NCT05143567.

All patients were on pathogenesis-targeted regimens (systemic glucocorticosteroids), etiotropic interventions (antiviral formulations), supportive symptomatic agents (antipyretics, mucoactive drugs, and bronchodilators), and supplementary oxygen insufflation according to the relevant guidelines [19]. Systemic anticoagulant agents were systematically administered to the entire study population. While patients presenting with normal body weight received standardized prophylactic doses, individuals with concomitant obesity underwent a guideline-directed 50% dose escalation to compensate for altered pharmacokinetic profiles. Consequently, non-obese participants received subcutaneous unfractionated heparin (UFH) at a dose of 5000 IU two to three times daily, or subcutaneous low-molecular-weight heparin (LMWH) at a fixed dose of 40 mg once daily. Conversely, within the obese cohort, the LMWH dosing regimen was adjusted to a weight-based therapeutic configuration of 1 mg/kg administered subcutaneously twice daily, whereas patients allocated to the UFH arm received a modified subcutaneous dose of 7500 IU three times daily. As for DOACs, rivaroxaban 20 mg was used once daily; with creatinine clearance of 30–49 mL/min, the dose of the drug was reduced to 15 mg once a day.

*Patient Cohort Stratification*

Based on the specific anticoagulant strategy deployed, the enrolled study population was stratified into three active therapeutic arms:

There were one hundred ninety patients with COVID-19 who received low-molecular-weight heparins, LMWH (group 1), median age 63 (56–70); one hundred twenty-three patients who received unfractionated heparin, UFH (group 2), median age 61 (50.5–68); fifty-seven patients who received direct oral anticoagulants, DOAC (group 3), median age 63 (52.5–71).

*COVID-19 Severity Classification [19]*

Mild cases of COVID-19 were characterized by a body temperature of less than 38 °C (100.4 °F), catarrhal symptoms such as cough, weakness, and sore throat, and the absence of criteria for a moderate or severe course of the disease.

Moderate cases included the following: a body temperature greater than 38 °C (100.4 °F), shortness of breath during physical exertion, a respiratory rate greater than 22 breaths per minute, oxygen saturation less than 95%, and changes on computed tomography (CT) typical of a viral infection. Laboratory tests included serum CRP levels greater than 10 mg/L.

Criteria for severe COVID-19 included decreased consciousness, agitation; unstable hemodynamics (systolic blood pressure less than 90 mmHg or diastolic blood pressure less than 60 mmHg, urine output less than 20 mL/h); respiratory rate greater than 30 breaths per minute, saturation less than 93%, PaO_2_/FiO_2_ ≤ 300 mmHg; CT changes in the lungs typical of viral infection; arterial lactate > 2 mmol/L; qSOFA > 2 points.

Extremely severe COVID-19 was characterized by acute respiratory distress syndrome (ARDS), characterized by persistent febrile fever. Acute respiratory failure developed, requiring respiratory support and requiring mechanical ventilation. This condition can be complicated by septic shock and multiple organ failure. CT scans revealed lung changes typical of critical viral infection or acute respiratory distress syndrome (ARDS).

*Baseline Clinical and Laboratory Screening*

Upon initial hospital admission, all patients underwent a meticulous baseline evaluation of clinical and anamnesis data alongside routine laboratory diagnostic panels, including complete blood counts, comprehensive metabolic profiles, and standard coagulation assays quantified via validated clinical laboratory methodologies.

*Blood Sampling, Processing, and Bio-banking Protocols*

To analyze target molecular markers, whole blood specimens (5 mL) were collected via venipuncture. Serum isolation was achieved through standardized centrifugation, after which serum aliquots were immediately cryopreserved and maintained at −80 °C until downstream analysis. Blood sampling was executed across three critical milestones: at baseline hospital admission; upon clinical deterioration necessitating immediate transfer to the intensive care unit (ICU), which also served as the primary clinical endpoint of the protocol; and at the conclusion of therapy immediately prior to hospital discharge. Human-specific enzyme-linked immunosorbent assay (ELISA) kits were used to measure phosphatidylserine serum level (catalog number CEB881Ge, Cloud-Clone Corp., Wuhan, China) and calreticulin serum level (catalog number CEB881Ge, Cloud-Clone Corp., China) in strict accordance with the manufacturer’s operational instructions.

In line with standard hospital protocols, serum levels of C-reactive protein (CRP), ferritin, and procalcitonin were monitored throughout the hospital stay to gauge the degree of inflammation.

*Vascular Ultrasonography Protocol*

To screen for venous thromboembolism, all participants underwent comprehensive duplex venous ultrasonography mapping both the deep and superficial venous systems of the lower extremities. This non-invasive vascular interrogation was systematically performed at three sequential time points: at baseline hospital admission, at the juncture of clinical worsening before ICU transfer, and at the termination of the treatment course upon hospital discharge.

*Pulmonary embolism*

In cases of suspected pulmonary embolism (PE), echocardiography and computed tomography pulmonary angiography (CTPA) were performed. Patients presenting with signs of PE upon admission were excluded from the study.

*Statistical Analysis*

Statistical processing of the clinical data was executed utilizing IBM SPSS Statistics software (version 26.0; IBM Corp., Armonk, NY, USA). The conformity of continuous variables to a normal distribution path was rigorously verified using the Kolmogorov–Smirnov test. Proportions and categorical variables were compared using Pearson’s chi-squared test or Fisher’s exact test where appropriate. Due to the predominantly non-Gaussian distribution profile of the analyzed metrics, continuous data were expressed as median (Me) accompanied by interquartile range [(IQR); (Q1–Q3)]. Consequently, non-parametric analytical frameworks were deployed for multi-group and intra-group comparative evaluations, specifically leveraging the Mann–Whitney (U) test, Wilcoxon signed-rank test, and Kruskal–Wallis (H) test, with statistical significance established at a two-tailed (*p*)-value (0.05). For the limited subsets exhibiting a Gaussian distribution path, corresponding parametric analogs were selectively applied to ensure analytical precision. No generative artificial intelligence was used in this manuscript to assist in study design, data collection, analysis or interpretation.

## 3. Results

*Baseline characteristics of patients*

At the initiation of the distinct anticoagulant regimens, the baseline study cohorts demonstrated high homogeneity and were fully comparable regarding chronological age, the clinical severity spectrum of SARS-CoV-2 infection, and routine laboratory parameters. The patients in the three groups were comparable in age (*p* = 0.213): median age in group 1 was 63 (56–70); median age in group 2 was 61 (50.5–68); median age in group 3 was 63 (52.5–71). Evaluation of the demographic gender profile revealed a consistent female predominance across all examined therapeutic arms: there were 55.3% females in group 1, 71.8% females in group 2, and 73.2% females in group 3 (*p* = 0.003). Information is given in Table 1.

COVID-19 severity was defined by the baseline lung computed tomography characteristics at admission. Mild COVID-19 upon admission was registered in 11.1% of cases in group 1, 4.8% cases in group 2, and 12.5% cases in group 3. Moderate COVID-19 was registered in 49.5% cases in group 1, 40.3% cases in group 2, and 33.9% cases in group 3. Severe COVID-19 was registered in 30.5% cases in group 1, 38.7% cases in group 2, and 46.5% cases in group 3. Critical COVID-19 was registered in 8.9% cases in group 1, 16.1% cases in group 2, and 7.1% cases in group 3 (*p* = 0.025).

Evaluation of baseline anamnestic data upon hospital admission revealed a dominant triad of concomitant metabolic and cardiovascular pathologies that demonstrated the highest prevalence across all examined cohorts, specifically comprising essential hypertension, obesity, and type 2 diabetes mellitus (Table 2).

*Comorbid Profiles and Baseline Anticoagulant Adjustments*

Statistically significant deviations in the prevalence of concomitant obesity and atrial fibrillation were selectively documented within the cohort managed with direct oral anticoagulants (DOACs; group 3). Patients allocated to this specific therapeutic arm were admitted to the inpatient facility while already undergoing long-term outpatient DOAC therapy for comorbid conditions, which was subsequently maintained throughout their hospitalization. For participants presenting with concomitant obesity, parenteral anticoagulant regimens were adjusted via a guideline-directed 50% dose escalation to compensate for altered pharmacokinetic profiles. Notably, chronic venous disease (CVD), a well-established independent risk factor for venous thromboembolism (VTE), was prevalent among individuals requiring acute parenteral anticoagulation, occurring in 8.0% of cases in group 1 (LMWH) and 16.0% of cases in group 2 (UFH; *p* = 0.03).

*Baseline Laboratory and Biomarker Profiling*

Comparative evaluation of laboratory matrices at the milestone of study enrollment revealed highly homogeneous and fully comparable baseline serum concentrations of calreticulin and phosphatidylserine, parallel to a uniform distribution of complete blood count parameters across all evaluated cohorts (Table 3). Characteristically, a profound upregulation of systemic acute-phase inflammatory reactants (specifically C-reactive protein [CRP] and ferritin) was consistently verified.

*Non-invasive and invasive mechanical ventilation*

**Figure 1:**
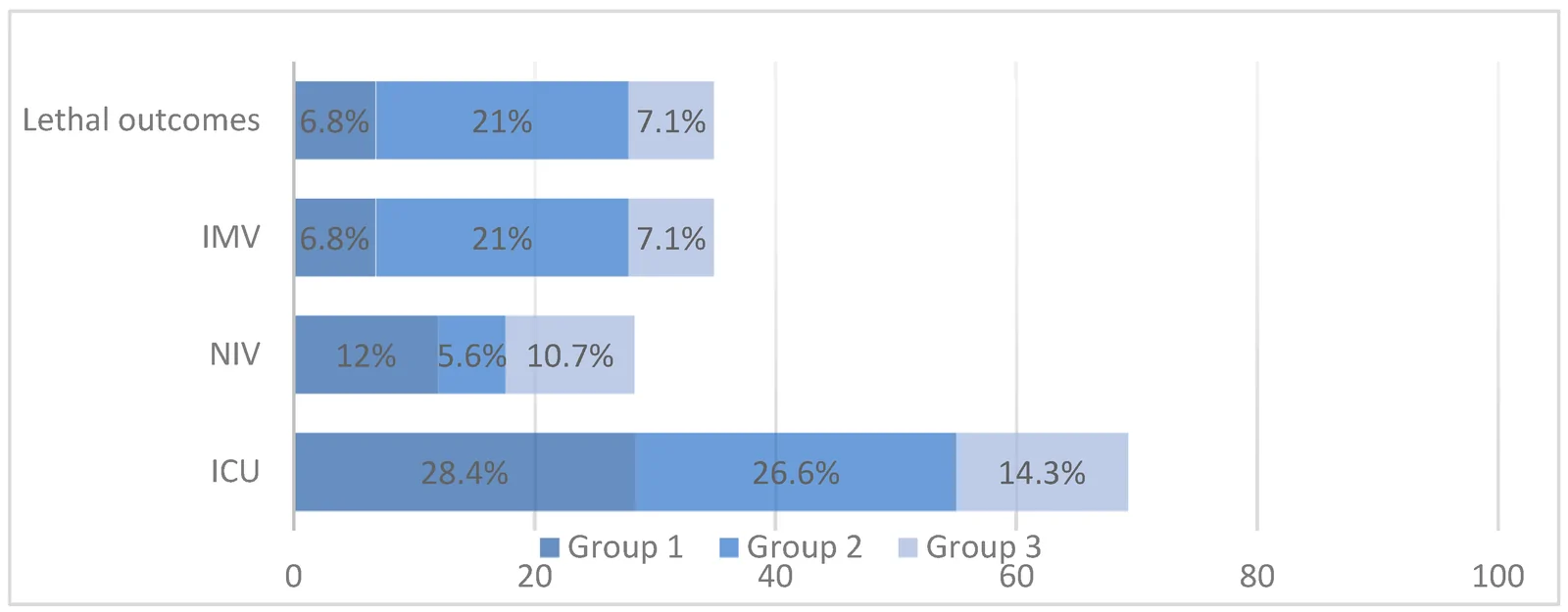
Utilization rates and distribution profiles of non-invasive and invasive mechanical ventilation within the intensive care unit setting across the active therapeutic arms. Note: ICU—intensive care unit. NIV—non-invasive ventilation. IMV—invasive mechanical ventilation.

The baseline data demonstrated that upon initiation of the distinct anticoagulant regimens, all active study cohorts exhibited high homogeneity and were fully comparable regarding chronological age, the clinical severity spectrum of SARS-CoV-2 infection, the specific COVID-19 treatment protocol administered, and routine laboratory parameters. The number of subjects requiring non-invasive ventilation (NIV) was lower in group 2 patients who received UFH (6% of cases). However, these patients were reported to eventually undergo invasive mechanical ventilation (IMV) significantly more often than group 1 patients who received LMWH and group 3 patients who received DOACs, 21% and 7%, respectively (*p* < 0.001) as presented in Figure 1. Crucially, the requirement for IMV transition served as an adverse clinical predictor that heavily compromised recovery trajectories, culminating in a markedly elevated mortality rate of 21.0% clustered within the UFH-treated cohort (group 2).

*Apoptosis Markers and Systemic Inflammatory Indices*

At the milestone of therapy termination, a statistically significant chronological surge in phosphatidylserine concentrations was exclusively documented within the cohort managed with low-molecular-weight heparin (LMWH; group 1), demonstrating an 18.4% increment from baseline parameters, shifting from 64.3 pg/mL to 76.15 pg/mL (*p* = 0.012) (Table 4). Characteristically, intense platelet activation drives the externalization and subsequent concentration increase in phosphatidylserine; this membrane phospholipid serves as a critical pro-apoptotic determinant possessing an exceptionally potent procoagulant catalytic surface. Subjects who received LMWH (group 1) were reported to have elevated end-of-treatment levels of calreticulin. Consequently, within the specific patient population characterized by the lowest incidence of venous thromboembolism (VTE; group 1), a coupled upregulation of both anti-apoptotic protectors and pro-apoptotic executioners was verified, indicating a highly regulated, non-destructive apoptotic equilibrium.

Conversely, no statistically discernible deviations in apoptosis marker kinetics were registered within the cohorts undergoing either unfractionated heparin (UFH) or direct oral anticoagulant (DOAC) treatments. Regarding systemic acute-phase reactants, C-reactive protein (CRP) levels demonstrated a consistent downward trend across all examined study cohorts (*p* = 0.135). However, the absolute residual concentrations of ferritin and procalcitonin (PCT) were significantly lower in patients who received targeted LMWH therapy compared to the alternative anticoagulant arms.

*VTE episodes*

**Figure 2:**
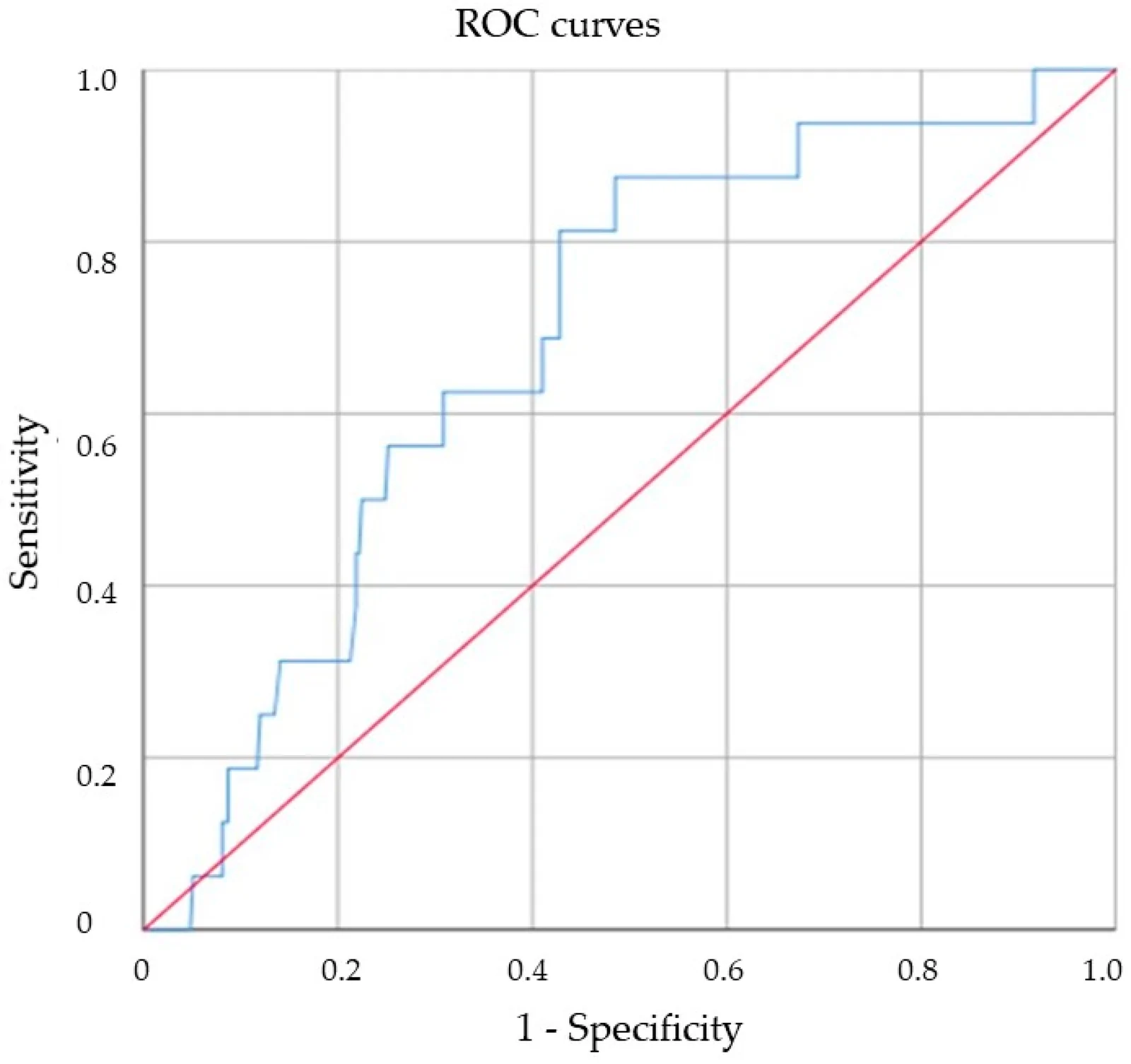
Receiver operating characteristic (ROC) curve analysis validating the multivariable model for venous thromboembolic risk prediction based on baseline phosphatidylserine level.

There were 1.6% of cases of isolated DVT in group 1, 6.5% cases in group 2, and 1.8% cases in group 3 (*p* = 0.031). A combination of DVT and PE was registered in 0.5% of cases in group 1, 6.5% cases in group 2, and 0% cases in group 3 (*p* = 0.003). Evaluation of venous thromboembolism rates unveiled a disproportionately high incidence of de novo pulmonary artery thrombosis—manifesting in the absolute absence of a primary embolic source within the lower extremities—accounting for 11.40% of cases in group 2 (unfractionated heparin; UFH) than 1.1% cases in group 1 and 0% cases in group 3 (*p* = 0.028). I.e., 11.4% of patients who received UFH were reported to experience suspected pulmonary artery thrombosis, i.e., pulmonary artery embolism in the absence of deep vein thrombosis or intracardiac thrombosis. This specific pathological phenomenon was systematically verified post-mortem through comprehensive autopsy protocols executed for the deceased study subjects (Figure 2). Concurrently, the patient population undergoing UFH therapy exhibited a significantly greater prevalence of both deep vein thrombosis (DVT) and secondary pulmonary embolism (PE) originating from localized lower extremity thrombi compared to the baseline outcomes documented in the low-molecular-weight heparin (LMWH; group 1) and direct oral anticoagulant (DOAC; group 3) cohorts.

*Evaluation of Hemorrhagic Complications*

Safety endpoint profiling regarding hemorrhagic complications revealed a significantly higher incidence of fatal major bleeding events exclusively within the patient population undergoing unfractionated heparin (UFH) therapy (group 2). Characteristically, these life-threatening adverse outcomes were predominantly represented by extensive gastrointestinal and massive bronchial hemorrhages: there were 0% major bleeding episodes in group 1 and group 3, and 4.9% major bleedings in group 2 (*p* = 0.004); there were 1.6% and 4.1% cases of clinically significant bleedings in group 1 and group 2, respectively (*p* = 0.246); there were 7.5%, 17.9%, and 5.3% cases of minor bleedings in group 1, group 2, and group 3, respectively (*p* = 0.025).

*Comprehensive Analysis of Venous Thromboembolic and Hemorrhagic Outcomes*

Within this specific critical care setting, a disproportionately high incidence of isolated pulmonary embolism (PE) occurring de novo—defined by the absolute absence of a primary embolic source within the lower extremities—was documented in 11.40% of cases and systematically verified post-mortem through comprehensive autopsy protocols executed for the deceased study subjects. Notably, antemortem duplex venous ultrasonography (DVUS) had been performed for these individuals, confirming the complete absence of a detectable peripheral thrombotic nidus. Patients treated with LMWH and DOAC were reported to develop the least number of VTE episodes. These cumulative findings demonstrate that the most lethal clinical complication was PE, particularly when developing in an isolated fashion with no verified DVT episodes.

Hemorrhagic rates were most frequent in patients treated with UFH, resulting in gastrointestinal and bronchial hemorrhages. Despite aggressive clinical interventions, these specific hemorrhagic events resulted in fatal outcomes. While the incidence of clinically relevant non-major bleeding was fully comparable across all examined cohorts, minor bleeding episodes occurred with a significantly greater frequency in patients receiving systemic UFH therapy.

*Receiver operating characteristic (ROC) curve analysis*

The binary logistic regression analysis and receiver operating characteristic (ROC) curve analysis demonstrated that the phosphatidylserine level over 62.75 pg/mL was associated with decreased odds of developing a VTE episode 1.033 (95% CI: 1.005–1.062; *p* = 0.02) (Figure 2). The area under the ROC curve (AUC) was 0.686 ± 0.06 (95% CI: 0.569–0.802), with a model significance of *p* = 0.012. Based on Youden’s index, the optimal PS cutoff threshold was determined to be 62.75 pg/mL. The direction of the analysis was descending; specifically, a PS level at or below the cutoff threshold predicted the development of VTE. The sensitivity and specificity of the method were 87.5% and 51.5%, respectively.

*Binary logistic regression*

The results of the binary logistic regression analysis are given in Table 5. The multivariate logistic regression model showed that the presence of chronic venous disease increased the odds of developing VTE by more than six times (OR 6.433, 95% CI 2.167–19.093, *p* = 0.001), invasive mechanical ventilation increased the odds of developing an episode of VTE by almost six times (OR 5.925, 95% CI 2.034–17.26, *p* = 0.001); and treatment with UFH increased the odds of developing VTE by more than three times (OR 3.542 CI 95% 1.149–10.916, *p* = 0.001).

## 4. Discussion

Patients diagnosed with severe SARS-CoV-2 infection exhibit a profoundly elevated incidence of vascular thrombosis; concurrently, these thromboembolic complications are intrinsically linked to adverse clinical trajectories and accelerated mortality rates. Global metadata derived from a comprehensive meta-analysis encompassing 42 independent clinical studies verified that the cumulative pooled incidence of venous thromboembolism reached 21.0%, with deep vein thrombosis (DVT) and pulmonary embolism (PE) accounting for 20.0% and 13.0% of cases, respectively [20]. Crucially, the prevalence of these occlusive events escalated exponentially within the intensive care unit (ICU) setting, surging to 31.0% for total VTE, 28.0% for DVT, and 19.0% for PE. In comparison, our single-center data revealed that the proportion of critically ill patients requiring transition to invasive mechanical ventilation (IMV) reached 21.0%, all of whom were selectively managed with systemic unfractionated heparin (UFH) (*p* < 0.001). Within our analyzed cohort, the requirement for IMV support served as a catastrophic clinical predictor associated with a 100% mortality rate. Conversely, patients undergoing low-molecular-weight heparin (LMWH) therapy demonstrated optimal survival profiles despite presenting with severe coronavirus-induced pulmonary injury, a protective trend primarily driven by the fact that the necessity for IMV deployment within this specific therapeutic arm was limited to only 6.0% of cases (*p* < 0.001).

Severe COVID-19 progression is characteristically driven by a hyperinflammatory immune-mediated counter-response, widely conceptualized as a cytokine storm. Nevertheless, the precise regulatory intersection where this hyperinflammatory cascade drives intravascular thrombogenesis remains to be fully elucidated. In the active study protocol, we sought to decipher the intricate pathobiochemical cross-talk linking systemic inflammation and macrovascular thrombosis within the pathophysiological framework of novel coronavirus infection, paying particular attention to the kinetics of apoptosis executioners and acute-phase reactants under the influence of distinct targeted anticoagulant regimens. Established biochemical evidence indicates that the molecular choreography of blood cell programmed death is fundamentally governed by the translational upregulation of pro-apoptotic executioners (Bax and Bak), parallel to the functional suppression of anti-apoptotic survival proteins (Bcl-2), which ultimately prompts the irreversible translocation and externalization of phosphatidylserine from the inner leaflet of the platelet plasma membrane to its outer catalytic surface [21]. Under the cytopathic impact of a “viral attack,” blood platelets turn into active executors of micro- and macrovascular catastrophes. Their robust activation triggers not only the assembly of the definitive fibrin-thrombus architecture but also the exhaustive exocytosis of an extensive pool of bioactive cargo that fuels and perpetuates the local inflammatory microenvironment [22]. This profound functional duality elevates blood platelets to the status of master regulators, orchestrating both the plasma coagulation cascade and the modulation of systemic immune-mediated inflammatory pathways.

In accordance with global metadata and consensus clinical expertise accumulated during the COVID-19 pandemic, our single-center data verified that all patient cohorts exhibited a profound baseline upregulation of systemic acute-phase inflammatory reactants—specifically C-reactive protein (CRP), ferritin, and procalcitonin (PCT). Conversely, the initial evaluation of target apoptosis indicators demonstrated high homogeneity, revealing fully comparable baseline levels across all investigated groups. Nevertheless, at the milestone of hospital discharge, a statistically significant chronological surge in phosphatidylserine concentrations was exclusively documented within the cohort managed with low-molecular-weight heparin (LMWH; group 1), presenting an 18.4% increment from baseline parameters, shifting from 64.3 pg/mL to 76.15 pg/mL (*p* = 0.012). Mechanistically, intense platelet activation drives the externalization of phosphatidylserine, which provides an exceptionally potent procoagulant catalytic surface.

However, of particular clinical relevance is the paradoxical disconnect wherein the markedly elevated end-of-treatment phosphatidylserine concentrations in the LMWH arm coexisted with a significantly lower incidence of deep vein thrombosis (DVT) compared to the unfractionated heparin (UFH) cohort, accounting for only 1.6% versus 6.5% of cases, respectively (*p* < 0.05). An identical protective trend was mirrored in pulmonary embolism (PE) rates, which were limited to 1.1% in group 1 compared to a disproportionately higher rate of 11.45% in group 2 (*p* < 0.05). This specific pathophysiological phenomenon can be rationalized by the translational limitation of routine antiplatelet regimens. While the administration of antiplatelet agents is fundamentally designed to suppress cellular activation pathways, it does not target or synergize with the plasma coagulation cascade. Consequently, these antiplatelet formulations are intrinsically incapable of attenuating or neutralizing the membrane-dependent procoagulant activity of activated platelets, a limitation effectively bypassed by the multifaceted pharmacodynamics of LMWH.

At the baseline milestone of study entry, all evaluated patient cohorts exhibited a profound, uniform upregulation of systemic acute-phase inflammatory reactants—specifically C-reactive protein (CRP), ferritin, and procalcitonin (PCT). However, the chronological kinetics of these laboratory metrics during the subsequent treatment course demonstrated distinct therapeutic deviations across the active arms. Our single-center data verified that a synchronized, substantial decline in both pro-inflammatory mediators and plasma coagulation parameters occurred exclusively at the end of therapy within the cohort managed with low-molecular-weight heparin (LMWH; group 1). This metabolic shift was associated with a significantly lower clinical incidence of venous thromboembolism throughout the entire hospitalization period compared to the outcomes documented in the unfractionated heparin (UFH) group (group 2). As seen in Table 4, in the LMWH group, the concentration of inflammatory markers, ferritin and procalcitonin, decreased at the end of inpatient treatment. This profound suppression of systemic hyperinflammation observed in the LMWH-treated cohort can possibly be rationalized by the extensive non-anticoagulant pleiotropic properties inherently possessed by certain parenteral glycosaminoglycans [23]. Beyond its core antithrombotic activity, LMWH exerts multi-targeted anti-inflammatory effects, including the competitive binding and neutralization of circulating pro-inflammatory cytokines, the functional inhibition of neutrophil chemotaxis, and the preservation of endothelial cell barrier structural integrity. Furthermore, LMWH formulations may elicit a potential antiviral impact, while simultaneously demonstrating a prolonged elimination half-life and a considerably lower biological potential to induce catastrophic bleeding events or heparin-induced thrombocytopenia compared to systemic UFH [24]. In strict alignment with these pharmacodynamic advantages, our clinical trial confirmed a significantly lower incidence of major bleeding events in the LMWH cohort compared to the UFH arm, accounting for 0.5% versus 4.9% of cases, respectively (*p* < 0.05). The high vulnerability to catastrophic hemorrhagic complications verified in patients receiving UFH can be structurally attributed to its complex chemical architecture, which is heavily dictated by the specific biological source of the raw material [25]. The molecular composition of mammalian-derived heparins exhibits substantial structural heterogeneity regarding the uronic acid residues within the glycosaminoglycan chains. Existing preclinical evidence indicates that bovine-derived heparin formulations demonstrate compromised therapeutic efficacy and trigger a significantly higher rate of bleeding complications compared to porcine-derived alternatives [26]. The lower phosphatidylseline levels in group 3 patients on DOAC could be possibly explained by the pre-existing protective benefit and pleiotropic effects of DOACs. However, the exact explanation cannot be comprehensively provided due to a limited number of patients on DOACs in this study.

The study has several limitations. Unfortunately, due to collecting specimens in a real-life COVID-19 hospital, we were unable to monitor anti-Xa activity during the admission. Also, the patients were not comparable in terms of sex distribution; however, this discrepancy was characteristic of real-world practice settings. Other limitations included a single-center design with limited generalizability, non-random treatment allocation, confounding by indication, unequal group sizes, and baseline differences in disease severity and comorbidity, as well as lack of external validation.

## 5. Conclusions

Biomarker profiling and multi-group comparative analytics revealed distinct laboratory profiles across the analyzed anticoagulant regimens. Specifically, a significant upregulation of both calreticulin and phosphatidylserine concentrations was documented within the patient population managed with low-molecular-weight heparin (LMWH; group 1). Inter-group comparative evaluations verified that end-of-treatment calreticulin levels in the LMWH cohort exceeded those observed in alternative arms (LMWH versus UFH: *p* < 0.001; LMWH versus DOACs: *p* = 0.024), whereas no statistically significant differences were registered between the UFH and DOAC populations (*p* = 0.764). An identical statistical distribution pattern was mirrored in phosphatidylserine kinetics, where group 1 metrics outpaced the alternative regimens (LMWH versus UFH: *p* = 0.021; LMWH versus DOACs: *p* = 0.005), while the UFH and DOAC cohorts demonstrated complete homogeneity (*p* = 0.913).

Regarding systemic acute-phase reactants, chronological monitoring indicated that C-reactive protein (CRP) levels shifted downward consistently across all examined cohorts (*p* = 0.135). However, the absolute residual concentrations of ferritin and procalcitonin (PCT) were lower at discharge in the LMWH arm. Pairwise multi-group analytics confirmed a profound suppression of ferritin in group 1 compared to the UFH arm (LMWH versus UFH: *p* < 0.001), whereas differences against DOACs did not reach the significance threshold (LMWH versus DOACs: *p* = 0.283; UFH versus DOACs: *p* = 1.0). This trend was further observed in PCT kinetics, where LMWH induced a markedly greater down-regulation compared to both alternative strategies (LMWH versus UFH: *p* < 0.001; LMWH versus DOACs: *p* < 0.001; UFH versus DOACs: *p* = 1.0). Consequently, these findings suggest the premise that LMWH formulations exert more potent pleiotropic anti-inflammatory and systemic anticoagulant activities within this infectious framework compared to alternative ACT protocols.

The study protocol formulated a prognostic model establishing that an elevated baseline phosphatidylserine level exceeding 62.75 pg/mL served as an independent protective factor within the LMWH cohort. This biological threshold mitigates the overall odds of subsequent venous thromboembolism (VTE) development, yielding a protective odds ratio (OR) of 0.968 with a 95% confidence interval (CI) of 0.942–0.995 (*p* = 0.02).

## Figures and Tables

**Table 1:** Clinical and anamnestic characteristics of the compared groups [Median (IQR)].

Parameter	Group 1— LMWH (*n* = 190)	Group 2— UFH (*n* = 123)	Group 3— DOAC (*n* = 57)	*p*-Value
Age	63 (56–70)	61 (50.5–68)	63 (52.5–71)	0.213
Sex n (%)				0.003
-male	85 (4.7)	35 (28.2)	15 (26.8)	
-female	105 (55.3)	89 (71.8)	41 (73.2)	
Severity COVID-19 according to the extent of lung involvement on computed tomography, n (%)				0.025 (difference in the extent of viral lung involvement) *p* = 0.067 (comparability of the groups across each category of lung involvement)
-0	11 (5.8)	0 (0)	2 (3.6)	
-1	43 (22.6)	23 (18.5)	11 (19.6)	
-2	81 (42.6)	53 (42.7)	17 (30.4)	
-3	45 (23.7)	37 (29.8)	24 (42.9)	
-4	10 (5.3)	11 (8.9)	2 (3.6)	

*Note: Statistical analysis was performed using Kruskal–Wallis test and pairwise comparisons with Bonferroni correction. LMWH—low molecular weight heparin. UFH—unfractionated heparin. DOAC—direct oral anticoagulant.*

**Table 2:** Concomitant pathology in the study groups.

Parameter	Group 1— LMWH (*n* = 190), n (%)	Group 2— UFH (*n* = 123), n (%)	Group 3— DOAC (*n* = 57), n (%)	*p*-Value
Obesity	76 (40)	66 (53.2)	9 (16.1)	*p* < 0.001
Arterial hypertension	151 (79.5)	96 (77.4)	44 (78.6)	LMWH-UFH *p* = 0.764
Diabetes mellitus	59 (31.1)	43 (35)	15 (26.8)	LMWH-DOAC *p* = 0.024
Ischemic heart disease	48 (25.3)	26 (21)	19 (33.9)	UFH-DOAC *p* < 0.001
Atrial fibrillation	30 (15.8)	17 (13.7)	38 (66.7)	
History of oncology	22 (11.6)	9 (7.3)	5 (8.9)	0.91
Bronchial asthma	6 (3.2)	9 (7.3)	3 (5.4)	0.531
Chronic obstructive pulmonary disease	15 (7.9)	2 (1.6)	0 (0)	0.178
History of peptic ulcers	11 (5.8)	13 (10.5)	3 (5.4)	*p* < 0.001
History of myocardial infarction	16 (8.5)	9 (7.3)	5 (8.9)	UFH-LMWH *p* = 0.924
Chronic kidney disease	39 (20.5)	7 (5.6)	6 (10.7)	LMWH-DOAC *p* < 0.013
Chronic venous disease	16 (8.4)	19 (15.3)	2 (3.6)	UFH-DOAC *p* = 0.017

*Note: Statistical analysis was performed using Kruskal–Wallis test and pairwise comparisons with Bonferroni correction. LMWH—low molecular weight heparin. UFH—unfractionated heparin. DOAC—direct oral anticoagulant.*

**Table 3:** Baseline laboratory parameters (Me(Q1–Q3)).

Parameter (Reference Value)	Group 1— LMWH (*n* = 190)	Group 2— UFH (*n* = 123)	Group 3— DOAC (*n* = 57)	*p*-Value
Calreticulin (4.73–15.59 pg/mL)	4.87 (2.7–7.96)	3.25 (2.13–5.35)	3.74 (2.18–6.38)	0.142
Phosphatidylserine (17.24–96.70 pg/mL)	63.3 (42.45–86)	63.7 (43.75–91)	57.1 (42.2–85.9)	0.489
C-reactive protein; (≤5 mg/L)	57.8 (24–101.3)	66.3 (38–126.9)	47.7 (17.2–104.4)	0.412
Ferritin (10–200 mcg/L)	738 (210–967)	848 (596–953)	432.5 (278–884)	0.371
Procalcitonin (≤0.5 ng/mL)	0.357 (0.348–0.469)	0.097 (0.07–0.173)	0.055 (0.04–0.145)	0.467

*Note: Statistical analysis was performed using Kruskal–Wallis test and pairwise comparisons with Bonferroni correction. LMWH—low molecular weight heparin. UFH—unfractionated heparin. DOAC—direct oral anticoagulant.*

**Table 4:** End-of-treatment laboratory parameters and biomarker distribution across the active therapeutic cohorts (median (Q1–Q3).

Parameter (Reference Value)	Group 1— LMWH (*n* = 190)	Group 2— UFH (*n* = 123)	Group 3— DOAC (*n* = 57)	*p*-Value
Calreticulin (4.73–15.59 pg/mL)	5.26 (2.89–12.95)	2.81 (1.01–4.51)	3.38 (1.79–6.78)	*p* < 0.001 LMWH-UFH *p* < 0.001 LMWH-DOAC *p* = 0.024 UFH-DOAC *p* = 0.764
Phosphatidylserine (17.24–96.70 pg/mL)	76.15 (56.25–110.55)	55.75 (43.2–91.6)	52.75 (42.6–86.4)	*p* = 0.001 LMWH-UFH *p* = 0.021 LMWH-DOAC *p* = 0.005 UFH-DOAC *p* = 0.913
C-reactive protein; (≤5 mg/L)	6.8 (1.7–15)	7.6 (4.05–13.35)	9.1 (4.55–19.65)	*p* = 0.135
Ferritin (10–200 mcg/L)	364 (324–497)	578.2 (367–986)	579 (402–952)	*p* < 0.001 LMWH-UFH *p* < 0.001 LMWH-DOAC *p* = 0.283 UFH-DOAC *p* = 1
Procalcitonin (≤0.5 ng/mL)	0.024 (0.004–0.364)	0.166 (0.005–0.274)	0.262 (0.045–0.385)	*p* < 0.001 LMWH-UFH *p* < 0.001 LMWH-DOAC *p* < 0.001 UFH-DOAC *p* = 1

*Note: Statistical analysis was performed using Kruskal–Wallis test and pairwise comparisons with Bonferroni correction. LMWH—low molecular weight heparin; UFH—unfractionated heparin; DOAC—direct oral anticoagulant.*

**Table 5:** Regression analysis of risk factors for developing VTE episodes (median (Q1–Q3).

Parameter (Reference Value)	Univariate Analysis		Multivariate Analysis	
	OR (95% CI)	*p*-Value	OR (95% CI)	*p*-Value
Age	1.02 (0.982–1.06)	0.296	-	-
Sex		0.206	-	-
-male	0.482 (0.155–1.495)			
-female	2.075 (0.669–6.435)			
BMI	1.192 (1.039–1.367)	0.012	1.179 (0.999–1.391)	0.051
Severity COVID-19 according to the extent of lung involvement on computed tomography:				
-1	0.751 (0.212–2.664)	0.658	-	-
-2	0.543 (0.189–1.555)	0.255	-	-
-3	2.629 (1.014–6.88)	0.047	1.768 (0.598–5.223)	0.303
-4	0.882 (0.112–6.94)	0.905	-	-
ICU stay	3.926 (1.502–10.267)	0.005	0.769 (0.087–6.794)	0.814
Severity of COVID-19				
-Mild	1.294 (0.284–5.891)	0.739	-	-
-Moderate	0.148 (0.034–0.655)	0.012	0.244 (0.049–1.223)	0.086
-Severe	0.897 (0.329–2.447)	0.832	-	-
-Extremely severe (critical)	7.733 (2.855–20.945)	<0.001	2.452 (0.573–10.505)	0.227
Comorbidity				
-Ischemic heart disease	1.968 (0.74–5.236)	0.175	-	-
-Prior myocardial infarction	0.696 (0.089–5.438)	0.73	-	-
-Atrial fibrillation	2.436 (0.831–7.14)	0.105	-	-
-Arterial hypertension	2.24 (0.504–9.954)	0.289	-	-
-Prior stroke	-	0.999	-	-
-Rheumatic heart disease	-	0.999	-	-
-Asthma	1.159 (0.146–9.231)	0.889	-	-
-COPD	1.235 (0.155–9.87)	0.842	-	-
-Peptic ulcer	1.635 (0.356–7.515)	0.528	-	-
-Diabetes mellitus	0.893 (0.307–2.595)	0.835	-	-
-Glucocorticoid-induced diabetes	1.485 (0.324–6.814)	0.611	-	-
-CKD	0.347 (0.045–2.666)	0.309	-	-
-Peripheral arterial disease	-	0.999	-	-
-Chronic venous disease	8.91 (3.264–24.326)	<0.001	6.433 (2.167–19.093)	0.001
-Prior VTE	-	0.998	-	-
-Anemia	-	0.999	-	-
-Oncology	0.531 (0.069–4.112)	0.545	-	-
-Prior trauma	-	0.999	-	-
-Prior coronary artery stenting	-	0.999	-	-
Invasive mechanical ventilation	9.353 (3.478–25.153)	<0.001	5.925 (2.034–17.26)	0.001
Non-invasive ventilation	0.533 (0.069–4.125)	0.547	-	-
Treatment with LMWH	0.255 (0.082–0.79)	0.018	0.882 (0.092–8.463)	0.914
Treatment with UFH	5.645 (1.964–16.223)	0.001	3.542 (1.149–10.916)	0.028
Treatment with DOAC	0.318 (0.041–2.436)	0.27	-	-

*Note: ICU—intensive care unit. COPD—chronic obstructive pulmonary disease. CKD—chronic kidney disease. VTE—venous thromboembolism. LMWH—low molecular weight heparin. UFH—unfractionated heparin. DOAC—direct oral anticoagulant.*

## Data Availability

The original contributions presented in this study are included in the article. Further inquiries can be directed to the corresponding authors.

## Supplementary Materials

The following supporting information can be downloaded at: https://www.mdpi.com/article/10.3390/life16101693/s1, Table S1: STROBE Statement.

## Abbreviations

The following abbreviations are used in this manuscript:

CRP	C-reactive protein
DOAC	Direct oral anticoagulants
DVT	Deep vein thrombosis
IMV	Invasive mechanical ventilation
LMWH	Low molecular weight heparins
NIV	Non-invasive ventilation
PCT	procalcitonin
PE	Pulmonary embolism
VTE	Venous thromboembolism
UFH	Unfractionated heparin

## References

[1] Seliverstov E.I., Lobastov K.V., Ilyukhin E.A., Apkhanova T.V., Akhmetzyanov R.V., Akhtyamov I.F., Barinov V.E., Bakhmetiev S.A., Belov M.V., Bobrov S.A. (2023). Prevention, diagnosis and treatment of deep vein thrombosis. Russian experts consensus. Flebologiya.

[2] Agapov A.B., Kalinin R.E., Mzhavanadze N.D., Povarov V.O., Nikiforov A.A., Snegur S.V., Suchkov I.A. (2024). Anticoagulation prophylaxis in patients with coronavirus infection. Nauka Molod..

[3] Ferrandis R., Escontrela B., Ferrando C., Hernández M., Herrera J., Hidalgo F., Librero J., Llau J., Martínez A., Pajares A. (2023). Efficacy of thromboprophylaxis with low-molecular-weight heparin in critical patients with COVID-19: An observational, prospective, and multicenter study. Rev. Esp. Anestesiol. Reanim..

[4] Borodina I.A., Selezneva I.A., Borisova O.V., Baldina O.A., Gil’mIyarova F.N., Gusyakova O.A., Kolot’yEva N.A. (2021). Blood groups and secretory status in COVID-19. Nauka Molod..

[5] Zhou F., Yu T., Du R., Fan G., Liu Y., Liu Z., Xiang J., Wang Y., Song B., Gu X. (2020). Clinical course and risk factors for mortality of adult inpatients with COVID-19 in Wuhan, China. Lancet.

[6] Connors J.M., Levy J.H. (2020). COVID-19 and its implications for thrombosis and anticoagulation. Blood.

[7] Xu Z., Shi L., Wang Y., Zhang J., Huang L., Zhang C., Liu S., Zhao P., Liu H., Zhu L. (2020). Pathological findings of COVID-19 associated with acute respiratory distress syndrome. Lancet Respir. Med..

[8] Lind S.E. (2021). Phosphatidylserine is an overlooked mediator of COVID-19 thromboinflammation. Heliyon.

[9] Wang J., Yu C., Zhuang J., Qi W., Jiang J., Liu X., Zhao W., Cao Y., Wu H., Qi J. (2022). The role of phosphatidylserine on the membrane in immunity and blood coagulation. Biomark. Res..

[10] Kimani S.G., Geng K., Kasikara C., Kumar S., Sriram G., Wu Y., Birge R.B. (2014). Contribution of defective PS recognition and efferocytosis to chronic inflammation and autoimmunity. Front. Immunol..

[11] Love M., Behrens-Bradley N., Ahmad A., Wertheimer A., Klotz S., Ahmad N. (2022). Plasma levels of secretory calreticulin among HIV infected patients in Bauchi State. J. AIDS HIV Treat..

[12] Michalak M. (2024). Calreticulin: Endoplasmic reticulum Ca^2+^ gatekeeper. J. Cell. Mol. Med..

[13] Fischer C.R., Mikami M., Minematsu H., Nizami S., Lee H.G., Stamer D., Patel N., Soung D.Y., Back J., Song L. (2017). Calreticulin inhibits inflammation-induced osteoclastogenesis and bone resorption. J. Orthop. Res..

[14] Kuwabara K., Pinsky D.J., Schmidt A.M., Benedict B., Brett B., Ogawa S., Broekman M.J., Marcus A.J., Sciacca R.R., Michalak M. (1995). Calreticulin, an antithrombotic agent which binds to vitamin K-dependent coagulation factors, stimulates endothelial nitric oxide production, and limits thrombosis in canine coronary arteries. J. Biol. Chem..

[15] Colling M.E., Tourdot B.E., Kanthi Y. (2021). Inflammation, infection, and venous thromboembolism. Circ. Res..

[16] Kaltenmeier C., Yazdani H.O., Morder K., Geller D.A., Simmons R.L., Tohme S. (2021). Neutrophil extracellular traps promote T cell exhaustion in the tumor microenvironment. Front. Immunol..

[17] Hu S., Lee H., Zhao H., Ding Y., Duan J. (2022). Inflammation in cerebral venous thrombosis. Front. Neurol..

[18] Valenzuela E.D., Mercado P., Pairumani R., Medel J.N., Petruska E., Ugalde D., Morales F., Eisen D., Araya C., Montoya J. (2021). Cardiac function during COVID-19 intensive care unit hospitalisation-deformation analysis and outcomes. Eur. Heart J. Cardiovasc. Imaging.

[19] Ministry of Health of the Russian Federation (2023). Temporary Clinical Guidelines. Prevention, Diagnosis and Treatment of Novel Coronavirus Infection (COVID-19).

[20] Xie D., Xue X., Qiao W., Wang J., Meng L., Ye Z., Li D., Sun Y. (2020). Thromboembolism risk of COVID-19 is high and associated with a higher risk of mortality: A systematic review and meta-analysis. EClinicalMedicine.

[21] Soto M., Palomo I., Ocqueteau M., Coetzee L.-M., Astudillo S., Aranda E., Mezzano D., Pereira J. (2002). Platelet aging in vivo is associated with activation of apoptotic pathways: Studies in a model of suppressed thrombopoiesis in dogs. Thromb. Haemost..

[22] Lippi G., Plebani M., Henry B.M. (2020). Thrombocytopenia is associated with severe coronavirus disease 2019 (COVID-19) infections: A meta-analysis. Clin. Chim. Acta.

[23] Walenga J.M., Jackson C.M., Kessler C.M. (2011). Low molecular weight heparins differ substantially: Impact on developing biosimilar drugs. Semin. Thromb. Hemost..

[24] Mousa S.A., Fareed J., Iqbal O., Kaiser B. (2004). Tissue factor pathway inhibitor in thrombosis and beyond. Methods Mol. Biol..

[25] Kalinin R.E., Suchkov I.A., Agapov A.B. (2017). Efficacy of different options for anticoagulant therapy for deep vein thrombosis of the lower extremities in routine clinical practice. Flebologiya.

[26] Tovar A.M.F., Teixeira L.A.C., Rembold S.M., Leite M., Lugon J.R., Mourão P.A. (2013). Bovine and porcine heparins: Different drugs with similar effects on human haemodialysis. BMC Res. Notes.

