# Supplementary material for: Biomarkers of Apoptosis and Systemic Inflammation in Patients with COVID-19 Undergoing Targeted Anticoagulant Therapy
Source: Life (Basel). 2026 Oct 9;16(10):1693. doi: 10.3390/life16101693 (PMC13706557; doi:10.3390/life16101693)
Supplement: Supplementary file 1 [file life-16-01693-s001.zip › life-4536537-supplementary.pdf]

Table S1. STROBE Statement—Checklist of items that should be included in reports of ***cohort studies***

	Item No	Recommendation
**Title and abstract**	1	(*a*) (*a*) Indicate the study's design with a commonly used term in the title or the abstract<br>prospective cohort study
		(*b*) Provide in the abstract an informative and balanced summary of what was done and what was found<br>provided in the abstract (page 1)
**Introduction**		
Background/rationale	2	Explain the scientific background and rationale for the investigation being reported<br>Explained in Introduction on pages 1 to 3
Objectives	3	State specific objectives, including any prespecified hypotheses<br>Provided on page 3
**Methods**		
Study design	4	Present key elements of study design early in the paper<br>Presented in Material and methods on page 4
Setting	5	Describe the setting, locations, and relevant dates, including periods of recruitment, exposure, follow-up, and data collection<br>Presented in Material and methods on page 4
Participants	6	(*a*) Give the eligibility criteria, and the sources and methods of selection of participants. Describe methods of follow-up<br>Presented in Material and methods on page 4
		(*b*) For matched studies, give matching criteria and number of exposed and unexposed
Variables	7	Clearly define all outcomes, exposures, predictors, potential confounders, and effect modifiers. Give diagnostic criteria, if applicable<br>Presented in Material and methods on pages 4 and 5
Data sources/ measurement	8*	For each variable of interest, give sources of data and details of methods of assessment (measurement). Describe comparability of assessment methods if there is more than one group<br>Presented in Material and methods on pages 4 and 5
Bias	9	Describe any efforts to address potential sources of bias<br>Presented in Material and methods on pages 4 and 5
Study size	10	Explain how the study size was arrived at<br>Presented in Material and methods on page 4
Quantitative variables	11	Explain how quantitative variables were handled in the analyses. If applicable, describe which groupings were chosen and why<br>Presented in Material and methods on page 6
Statistical methods	12	(*a*) Describe all statistical methods, including those used to control for confounding
		(*b*) Describe any methods used to examine subgroups and interactions
		(*c*) Explain how missing data were addressed
		(*d*) If applicable, explain how loss to follow-up was addressed
		(*e*) Describe any sensitivity analyses<br>Statistical methods are presented in Material and methods on page 6
**Results**		
Participants	13*	(a) Report numbers of individuals at each stage of study—eg numbers potentially

		eligible, examined for eligibility, confirmed eligible, included in the study, completing follow-up, and analysed
		(b) Give reasons for non-participation at each stage
		(c) Consider use of a flow diagram<br>Information on participants is provided in Results on pages 6 and 7
Descriptive data	14*	(a) Give characteristics of study participants (eg demographic, clinical, social) and information on exposures and potential confounders
		(b) Indicate number of participants with missing data for each variable of interest
		(c) Summarise follow-up time (eg, average and total amount)<br>Information on participants is provided in Results on pages 6 and 7
Outcome data	15*	Report numbers of outcome events or summary measures over time<br>Information is provided in Results on page 8 to 11
Main results	16	(*a*) Give unadjusted estimates and, if applicable, confounder-adjusted estimates and their precision (eg, 95% confidence interval). Make clear which confounders were adjusted for and why they were included
		(*b*) Report category boundaries when continuous variables were categorized
		(*c*) If relevant, consider translating estimates of relative risk into absolute risk for a meaningful time period<br>Information, including calculation of OR is provided in Results on pages 11 to 12
Other analyses	17	Report other analyses done—eg analyses of subgroups and interactions, and sensitivity analyses<br>Information, including ROC analysis is provided in Results on pages 13 to 14
**Discussion**		
Key results	18	Summarise key results with reference to study objectives<br>Presented on pages 14 to 16 in Discussion
Limitations	19	Discuss limitations of the study, taking into account sources of potential bias or imprecision. Discuss both direction and magnitude of any potential bias<br>Limitations are presented on page 16 in Discussion
Interpretation	20	Give a cautious overall interpretation of results considering objectives, limitations, multiplicity of analyses, results from similar studies, and other relevant evidence<br>Final interpretation is given in Conclusions on pages 16 and 17
Generalisability	21	Discuss the generalisability (external validity) of the study results<br>External validation has not been performed, which is reflected in the Limitations area in Discussion section
**Other information**		
Funding	22	Give the source of funding and the role of the funders for the present study and, if applicable, for the original study on which the present article is based<br>The information is given on page 17. This research received no external funding.

*Give information separately for exposed and unexposed groups.

Note: An Explanation and Elaboration article discusses each checklist item and gives methodological background and published examples of transparent reporting. The STROBE checklist is best used in conjunction with this article (freely available on the Web sites of PLoS Medicine at http://www.plosmedicine.org/, Annals of Internal Medicine at http://www.annals.org/, and Epidemiology at http://www.epidem.com/). Information on the STROBE Initiative is available at http://www.strobe-statement.org.
